# Pain in Parkinson's Disease Associated with *COMT* Gene Polymorphisms

**DOI:** 10.1155/2014/304203

**Published:** 2014-03-11

**Authors:** Wanjun Li, Yongqian Chen, Bowen Yin, Limei Zhang

**Affiliations:** Department of Neurology, Second Hospital of Harbin Medical University, Harbin, Heilongjiang Province 150086, China

## Abstract

*Background*. PD patients present high incidence of pain with unknown pathogenesis. *Objective*. We investigated the relation of *COMT *polymorphisms *rs4633* and *rs6267* with PD pain. *Subjects and Methods*. One hundred PD patients and 105 controls were evaluated with simplified Mc GILL pain scale and VAS scale. PD patients were assessed with H&Y grade, UPDRS score, and HAMD scale. Polymorphisms *rs4633 * and *rs6267* were detected by PCR and direct sequencing. *Results*. Fifty-seven percent of PD patients experienced pain, consisting of PD-related pain (64.91%) (the majority was dystonia pain) and non-PD-related pain (35.09%) (psychogenic pain was most frequent). The frequency of *rs6267* genotype “GT/TT” and allele “T” was higher in PD pain. No difference was observed in frequencies of *rs4633* between PD pain and without pain. UPDRS and depression score were higher in PD pain. The onset age was earlier in PD-related pain (57.43 ± 19.71) than non-PD-related pain (63.36 ± 6.88). *Conclusion*. PD patients possess a high prevalence of pain. Dystonia pain was the most frequent type of PD-related pain. *COMT* gene *rs6267* allele “T” associated with PD pain. PD pain was influenced by disease severity and depression. PD onsets earlier in patients with PD-related pain than non-PD-related pain.

## 1. Introduction 

Patients with Parkinson's disease (PD) present a reduction of pain threshold [1] and high incidence of pain [2, 3]. The pathogenesis of pain remains unclear; it may be affected by genetic factors. The pain and analgesic sensitivity tests found that genetic factors accounted for 28–76% [4]; pain response has been confirmed to associate with gene polymorphism through pressure stimulation, thermal stimulation, electrical stimulation, and ischemia [5]. More than 600 genes are associated with pain. PD is characterized by reduced dopamine. At the same time, monoamine (norepinephrine and 5-hydroxytryptamine) showed abnormal metabolism [6]. While monoamine is an important neurotransmitter that regulates pain, PD pain is partially the result of monoamine system abnormalities [7].

Catechol-O-methyltransferase (*COMT*) is an important metabolic enzyme of monoamine and its gene polymorphism is related to the occurrence of pain [8–10].* COMT* gene mutation may induce decrease of* COMT* enzyme thermostability and activity and then degradation of dopamine decrease. These lead to overactivation of dopaminergic system, reducing endogenous enkephalin levels, and then compensatory increase of *μ*-opioid receptor capacity, increasing glutamate, and P substances release, enhancing afferent nociceptive impulse, and ultimately tolerance to pain decreased and pain sensitivity increased [11, 12]. Diatchenko et al. have detected the haplotypes consisting of* COMT rs6269*,* rs4633, rs4818,* and* rs4680* polymorphisms: low-pain-sensitive haplotype (LPS: G_C_G_G), average-pain-sensitive haplotype (APS: A_T_C_A), and high-pain-sensitive haplotype (HPS: A_C_C_G).* COMT* activity in low-pain-sensitive haplotype is 4.8 times of the average-pain-sensitive haplotype and 11.4 times of the high-pain-sensitive haplotype [13, 14]. Since these haplotypes may be associated with susceptibility to PD, also affect therapeutic effect of levodopa on PD, and produce complications [15], thus the role of* COMT* on PD pain is worthy of following with interest. Among the pain-associated genetic polymorphisms,* rs4633* showed allele C in the low- and high-pain-sensitive haplotypes and it did not change with the altered sensitivity of pain and thus its contribution to pain remains elusive. While* COMT rs6267*, adjacent to* rs4633,* was considered to associate with pain [16], there are no reports addressing these two polymorphisms on the PD pain. This study was performed to explore the correlation between* COMT* gene polymorphisms* rs6267* and* rs4633 *and PD pain.

In addition to genetic susceptibility, age, sex, depression, and UPDRS score, L-Dopa medication also affect the occurrence of pain [17, 18]. These factors together with gene may lead to pain in PD. It was reported that female had lower pain threshold than male [19]. It may be the reason that the prevalence of pain was higher in female than male. Patients with depression showed disorders in 5-HT and norepinephrine pathway which were congenerous transmitters of pain. Likewise, L-Dopa medication interferes with monoamine systems. It makes up one of the pain reasons in PD. All of these indicate that pain is multifactorial and heterogeneity in PD patients. So these clinical factors were included in this study and we hope that it can provide further insight to PD pain and give evidence for treatment.

## 2. Subjects and Methods

One hundred patients, 51 males and 49 females, aged 34–85 years (average 65.84 ± 9.92), were included in this study. Patients were diagnosed as idiopathic PD according to the diagnosis criteria of Brain Bank of British Association of PD (UK), with MMSE score ≥ 24. No case had undergone stimulation of deep brain electrodes. PD patients had received L-Dopa for 0.5–9 years. The duration of L-Dopa medication was recorded as “0” if the patient did not receive L-Dopa. The disease severity was evaluated with Hoehn and Yahr (H&Y) grade and the unified PD rating scale (UPDRS), and mental state of patients was assessed with the Hamilton depression rating scale for depression. At the same time, 105 healthy aged volunteers were recruited as controls, including 63 males and 42 females, aged 28–81 years (average 60.32 ± 11.01), excluding PD and Parkinson's syndrome. Pain lasting for more than one month can be clearly identified as the accompanying symptom. Pain types were assessed with the simplified Mc GILL pain scale. The degree of pain was quantified by visual analogue 10-point scale (VAS). PD patients were divided to PD with pain and without pain according to this concept; based on the relation of pain with disease, PD patients with pain was classified as PD-related pain and non-PD-related pain according to the Ford classification [20].

Genomic DNA was extracted with phenol extraction method, and* COMT* gene polymorphism was determined with PCR and direct sequencing methods (sequencing was performed by SinoGenoMax, Chinese national human genome center, Beijing). The reaction system of PCR was totally 25 *μ*L, including 0.5 *μ*L DNA template, 0.5 *μ*L primers, 2 *μ*L dNTP, 2.5 *μ*L buffer, and 0.25 U Taq DNA polymerase. Annealing temperature was 60.9°C, and extension temperature was 72.0°C. Primers were designed as follows: forward 5′CAT TTC TGA ACC TTG CCC CTC 3′; reverse 5′CCT GTC CCA GAG CTG AGC ACC 3′ [21].

Data was analyzed by SPSS18.0. Binary logistic regression was used in assessing the correlation between* COMT* genotype and pain among PD patients, as well as in analyzing PD-related pain, and covariates were age, sex, onset age, duration of disease, duration of L-Dopa medication, and UPDRS. Allele frequencies were compared with chi-square test. The influencing factors of PD pain and PD-related pain were analyzed using analysis of variance of factorial design. *P* value was corrected by false discovery rate (FDR) (aF = PiN/i) or Bonferroni correction.

The study was approved by the local ethical committee, and all patients gave written informed consent.

## 3. Results

### 3.1. Clinical Data

Among the involved 100 PD patients, 57 cases (57%) reported pain and their average age was 65.47 ± 9.81 years, including 25 males (43.9%) and 32 females (56.1%); other 43 cases were not accompanied with pain, with an average age of 66.33 ± 10.17 years, including 26 males (60.5%) and 17 females (39.5%). In the control group, there were eight cases with pain (7.6%): arthralgia in five cases, sciatica in one case, gout in one case, and pain caused by surrounding tissue necrosis in one case.

**Figure 1:**
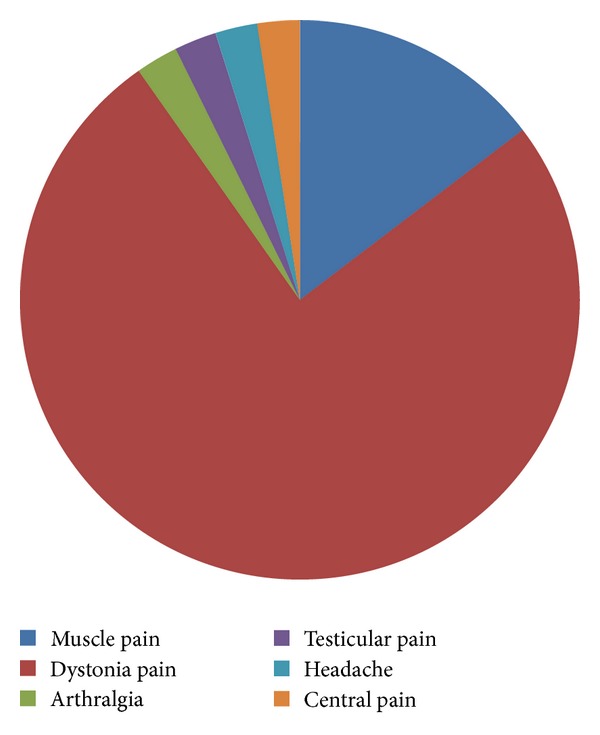
Category of PD-related pain.

**Figure 2:**
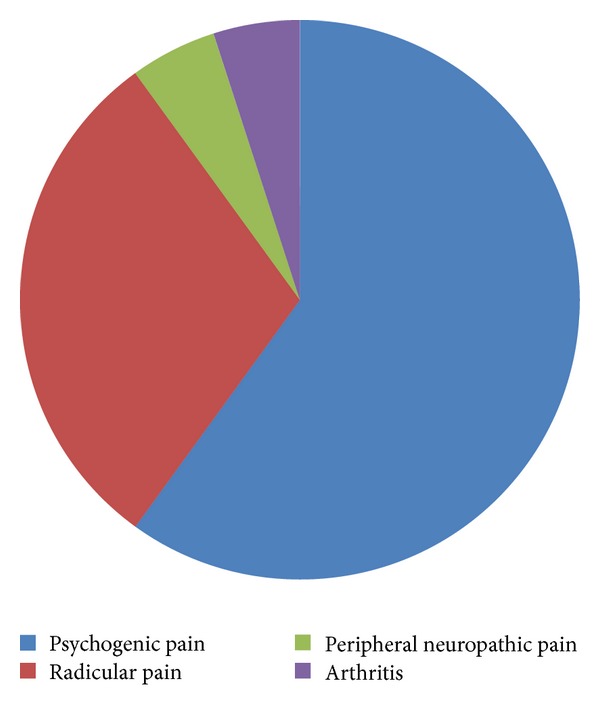
Category of non-PD-related pain.

Among the 57 PD patients who reported pain, 37 cases were PD-related pain (64.91%) (Figure 1): skeletal muscle pain in six cases, dystonia pain in 31 cases (hypermyotonia and/or reverse position), arthralgia in one case (no joint image changes), testicular pain in one case, headache in one case, and unexplained systemic unbearable pain in one case, which are considered central pain; the remaining 20 cases were non-PD-related pain (35.09%): psychogenic pain in 12 cases (60%), radicular pain in six cases, peripheral neuropathic pain in one case, and arthritis-caused joint pain in one case (Figure 2).

### 3.2. Distribution of* COMT rs4633* and* rs6267* in PD and Control Group

Ninety-seven PD patients and 105 controls obtained* COMT* gene* rs6267* and* rs4633* sequences. In PD group, genotype and allele frequencies are as follows: genotype of* rs4633* CC (0.485), CT (0.464), and TT (0.052); allele of* rs4633* C (0.716), T (0.284); genotype of* rs6267* GG (0.701), GT (0.268), and TT (0.031); allele G (0.835), T (0.165). Hardy-Weinberg equilibrium test in PD group was* rs4633* (*χ* ^2^ = 1.074, *P* = 0.584),* rs6267* (*χ* ^2^ = 0.014, *P* = 0.993). In the control group, frequency of genotypes was* rs4633*CC (0.619), CT (0.362), TT (0.019), allele of* rs4633* was C (0.800), T (0.200); genotype of* rs6267*GG (0.829), GT (0.143), TT (0.029), allele of* rs6267*G (0.900), T (0.100). Hardy-Weinberg equilibrium test was performed in control group:* rs4633* (*χ* ^2^ = 0.919, *P* = 0.632),* rs6267* (*χ* ^2^ = 1.494, *P* = 0.474). They all meet Hardy-Weinberg equilibrium.

### 3.3. Correlation between* COMT rs4633* and* rs6267* and PD Pain

The correlation between polymorphisms and pain were analyzed according to autosomal dominant and recessive mode of inheritance, respectively. The frequency of* rs6267* variant genotypes “GT/TT” (Table 1) and variant allele “T” (Table 2) was higher in PD patients with pain than patients without pain, if this polymorphism was autosomal dominant inherited; there were no statistic difference if rs6267 was autosomal recessive inherited, while* rs4633* genotype and allele frequencies showed no significant difference between PD patients with and without pain, whether it was autosomal dominant or recessive inherited.

### 3.4. Correlation between* COMT rs4633* and* rs6267* and PD-Related Pain

As to PD patients with pain (55 cases), 35 cases showed PD-related pain, 20 cases showed PD-unrelated pain (non-PD- related pain). The* rs6267* and* rs4633* genotype frequencies showed no significant difference between PD-related and non-PD-related pain group (Table 3), so the allele was not calculated.

### 3.5. Factors Influencing Pain in PD Patients

Clinical risk factors were included in this study; they are age and onset age of PD, course of disease, duration of L-Dopa medication, and depression and UPDRS scores (Table 4). Statistical analysis showed that UPDRS and depression scores were significantly different between PD patients with pain and without pain (after Bonferroni correction). There were no significant differences in the age, onset age of PD, disease duration, and duration of L-Dopa medication between the two groups.

Further analysis about PD-related pain, results showed that patients with PD-related pain displayed earlier onset age of PD than non-PD-related pain group (after Bonferroni correction). No statistic difference was found in age, duration of PD, duration of L-Dopa application, depression score, H&Y score, and UPDRS between these two group, and there was also no difference in duration of pain and VAS pain score (Table 5).

## 4. Discussion

Understanding the nonmotor symptoms of PD patients allows further attention on the pain. The prevalence of pain in general population is 0.1–0.3% [22] and increases in individuals aged *⩾*65 years [23]. In this study, 57% of PD patients complained about pain, with the prevalence being significantly higher than the control group (7.6%). Pain in PD patients presents a series of complex clinical manifestations, so that there is no uniform objective criterion for classification, which currently depends on the patient's description; in addition, several pain categories often overlap to each other. Our findings indicated that dystonia pain was dominant in PD-related pain (64.91%), followed by skeletal muscle pain. This was inconsistent with previously reported that musculoskeletal pain accounted for 70% and dystonia for 40% [24]. The difference may be explained by variations of the study population; patients gave vague descriptions of pain, and researchers gave different judgments towards the pain. The non-PD-related pain is primarily psychogenic pain; even though it is regarded that the pain is not linked to PD, the pain type is still different with pain in control group (while it is mainly joint pain in the control group). It indicates that non-PD-related pain still has flavor of PD and the criteria may be arbitrary which have been used to subdivide pain as PD-related and non-PD-related pain.

*COMT* gene* rs4633* polymorphism is associated with susceptibility to PD [25].* rs4633* polymorphism participates in variable pain-sensitive haplotype (LPS, APS, and HPS).* rs4633* showed allele C in LPS and HPS haplotypes, while it showed allele T in APS haplotype.* COMT* gene polymorphisms may be inherited through an autosomal dominant or recessive trait. In this study,* rs4633* genotype and allele frequencies showed no significant difference between PD patients with pain and without pain, either according to autosomal dominant or recessive mode of inheritance as well, and no difference was found about frequencies of* rs4633* genotypes between PD-related pain group and non-PD-related pain group. This suggests that* rs4633* is unrelated to pain in PD patients. The role of* rs4633* in different* COMT* haplotypes remains unclear and deserves further studies; at least it contributes little to the pain sensitivity.

*COMT* gene* rs6267* is missense mutation, which encodes the serine (Ser) instead of alanine (Ala). This missense mutation leads to change of RNA secondary structure; then* COMT* enzymatic activity decreased, thus being highly sensitive to pain [16]. In this study,* rs6267* variant genotype “GT/TT” and variant allele “T” were frequently distributed in PD patients with pain, comparing to patients without pain; it is based on autosomal dominant inherited trait. It indicates that* rs6267* associated to pain in PD. It confirmed that the low activity of* COMT* affects pain pathway. The mechanisms may be induced by abnormal metabolisms of monoamine in stratum [26], or spinal nociception pathway. On the other hand, there was no statistic difference if* rs6267* was inherited by recessive mode. This statistic result may be affected by sample size, so further investigation was needed in expanding the number of subjects.

It is reported that positive relation had been found between PD pain and sex, age, depression, and disease severity [27, 28], but the converse results are also reported [29, 30]. In order to exclude the influence of clinical factors on pain, we applied age, sex, onset age of PD, duration of disease, duration of L-Dopa medication, and UPDRS as covariates when statistic analysis was performed on* COMT* polymorphisms. Furthermore, we applied analysis of variance of factorial design to analyze the correlation between pain and clinical factors. The results showed that UPDRS scores were greater in PD group with pain (*P* < 0.05 after Bonferroni correction); thus it is regarded as a risk factor for PD pain. The higher UPDRS score indicated the higher prevalence of pain. UPDRS score indicates disease severity. The disease may induce pain through central and peripheral mechanisms. Central mechanism may be explained by the exacerbation of abnormal metabolism of monoamine, affecting the medial and lateral pain pathways. Peripheral mechanisms include peripheral neuropathy, muscle contraction, and fasciitis.

Chou [31] suggested that depressive patients may show pain within two years; while patients with a sense of pain may develop depression after two years. This is because 5-hydroxytryptamine and noradrenalin are the neurotransmitters for both pain and depression. Depressive patients may present a reduction of sensitivity to exogenous pain stimuli and an increase of sensitivity to endogenous pain [32]. Depression was more frequently seen in PD patients, and our findings showed that depression is a risk factor for PD pain.* COMT* gene polymorphism has been reported to be associated with depressive mood [21, 33]. There may be complex relation among PD, depression, and* COMT* gene.

As to the patients with pain, two group were identified as PD-related and non-PD-related pain. These two groups had no difference in frequencies of* COMT rs6267* and* rs4633*. The distributions were similar between PD-related and non-PD-related pain including age of patients, duration of PD, duration of L-Dopa application, depression score, H&Y grade, and UPDRS. There were no difference in pain duration and VAS pain score. The only difference occurs in onset age of PD (*P* = 0.031) and earlier onset age appeared in patients with PD-related pain. It means that the patients may be liable to experience PD-related pain, if he (she) gets PD in relative younger age. The reason is unclear; it needs more information to classify PD-related pain from non-PD-related pain.

In summary, 57% of PD patients experienced pain in this study, while 7.6% of controls had pain. 64.91% of patients with pain were classified as PD-related pain. Dystonia pain was the main part of PD-related pain; 35.09% was non-PD-related pain and psychogenic pain was most frequent.* COMT rs6267* genotype “GT/TT” and allele “T” were more frequent in PD patients with pain. Patients with pain showed higher UPDRS score and depression score compared with patients without pain. The patients with PD-related pain showed earlier onset age of PD.

## 5. Conclusion 

PD patients possess a high incidence of pain including PD-related pain and non-PD-related pain. Dystonia pain was the most frequent type of PD-related pain.* COMT* gene* rs6267* allele “T” associated with pain in PD patients. Pain in PD was influenced by disease severity and depression. PD onset age was in patients with PD-related pain than non-PD-related pain.

## Figures and Tables

**Table 1:** *COMT* genotype and PD with or without pain.

	PD with pain (*N* = 55)	PD without pain (*N* = 42)	AD OR (95% CI)	*P* value (AD)	AR OR (95% CI)	*P* value (AR)
*rs4633 *						
CC	25 (0.455)	22 (0.524)	0.776 (0.291–2.067)	0.612		
CT	27 (0.491)	18 (0.429)				
TT	3 (0.055)	2 (0.048)			1.582 (0.222–11.294)	0.648
*rs6267 *						
GG	33 (0.600)	35 (0.833)	0.216 (0.068–0.688)	0.010*		
GT	20 (0.364)	6 (0.143)				
TT	2 (0.036)	1 (0.024)			0.484 (0.028–8.487)	0.619

Binary logistic regression was used; covariates were age, sex, onset age, duration of disease, duration of L-Dopa medication, and UPDRS. *P < 0.05 after FDR correction (aF = 0.010 ∗ 8/2); *COMT:* catechol-O-methyltransferase, PD: Parkinson's disease, AD: autosomal dominant inheritance, AR: autosomal recessive inheritance, and OR: odds ratio.

**Table 2:** *COMT* allele and PD with or without pain.

	PD with pain	PD without pain	*χ* ^2^	*P* value
*rs4633 *				
C	77 (0.70)	62 (0.738)	0.340	0.560
T	33 (0.30)	22 (0.262)		
*rs6267 *				
G	86 (0.782)	76 (0.905)	5.227	0.022*
T	24 (0.218)	8 (0.095)		

Chi-square test, *P < 0.05. *COMT*: catechol-O-methyltransferase; PD: Parkinson's disease.

**Table 3:** *COMT* genotype and PD related pain.

	PD-related pain (*N* = 35)	Non-PD-related pain (*N* = 20)	AD OR (95% CI)	*P* value (AD)	AR OR (95% CI)	*P* value (AR)
*rs4633 *						
CC	14 (0.400)	11 (0.550)	0.458 (0.120–1.751)	0.254		
CT	19 (0.543)	8 (0.400)				
TT	2 (0.057)	1 (0.050)			1.156 (0.070–19.105)	0.919
*rs6267 *						
GG	22 (0.629)	11 (0.550)	1.384 (0.346–5.526)	0.646		
GT	13 (0.371)	7 (0.350)				
TT	0 (0)	2 (0.100)			4.509*E*9 (0.000)	0.999

Binary logistic regression was used; covariates were age, sex, onset age, duration of disease, duration of L-Dopa medication, and UPDRS. FDR correction was not performed for *P* > 0.05.

**Table 4:** Clinical factors on pain in PD patients.

	PD with pain (*N* = 55)	PD without pain (*N* = 42)	*F* value	*P*
Age (years)	65.44 ± 9.88	66.43 ± 10.27	0.232	0.631
Onset age (years)	59.58 ± 9.85	61.57 ± 10.75	0.897	0.346
Duration of PD (years)	5.85 ± 2.99	4.86 ± 3.18	2.506	0.117
Duration of medication (years)	3.47 ± 2.02	2.92 ± 2.24	1.647	0.202
Depression score	12.73 ± 11.12	7.12 ± 5.60	8.938	0.004*
UPDRS	46.27 ± 16.62	37.26 ± 15.08	7.580	0.007*

Analysis of variance of factorial design was used. Bonferroni correction was performed. *P < 0.05 (depression and UPDRS score). PD: Parkinson's disease; UPDRS: the unified Parkinson's disease rating scale.

**Table 5:** Factors between PD-related pain and non-PD-related pain.

	PD-related pain (N = 35)	Non-PD-related pain (N = 20)	F value	*P*
Age (years)	63.83 ± 10.95	68.25 ± 7.08	2.624	0.111
Onset age (years)	57.43 ± 19.71	63.36 ± 6.88	4.928	0.031*
Duration of PD (years)	6.43 ± 3.16	4.85 ± 2.43	3.726	0.059
Duration of medication (years)	3.86 ± 2.14	2.80 ± 1.61	3.669	0.061
Depression score	12.69 ± 12.14	12.80 ± 9.36	0.001	0.971
H&Y	2.27 ± 0.68	2.35 ± 0.81	0.148	0.702
UPDRS	46.51 ± 17.14	45.85 ± 16.09	0.020	0.888
Duration of pain (tears)	1.69 ± 1.09	1.69 ± 1.20	0.000	0.987
VAS pain score	5.00 ± 1.78	5.30 ± 1.95	0.337	0.564

Analysis of variance of factorial design was used. Bonferroni correction was performed. *P < 0.05 (onset age). PD: Parkinson's disease; UPDRS: the unified Parkinson's disease rating scale.

## References

[1] Marsala ZS, Tinazzi M, Vitaliani R (2011). Spontaneous pain, pain threshold, and pain tolerance in Parkinson’s disease. *Journal of Neurology*.

[2] Ha AD, Jankovic J Pain in Parkinson's disease. *Movement Disorders Journal*.

[3] Broen MPG, Braaksma MM, Patijn J, Weber WEJ (2012). Prevalence of pain in Parkinson’s disease: a systematic review using the modified QUADAS tool. *Movement Disorders*.

[4] Singewald N, Philippu A (1998). Release of neurotransmitters in the locus coeruleus. *Progress in Neurobiology*.

[5] Mogil JS (1999). The genetic mediation of individual differences in sensitivity to pain and its inhibition. *Proceedings of the National Academy of Sciences of the United States of America*.

[6] Prinz A, Selesnew LM, Liss B, Roeper J, Carlsson T (2013). Increased excitability in serotonin neurons in the dorsal raphe nucleus in the 6-OHDA mouse model of Parkinson’s disease. *Experimental Neurology*.

[7] Dellapina E, Gerdelat-Mas A, Ory-Magne F (2011). Apomorphine effect on pain threshold in Parkinson’s disease: a clinical and positron emission tomography study. *Movement Disorders*.

[8] Nackley AG, Diatchenko L (2010). Assessing potential functionality of catechol-*O*-methyltransferase (COMT) polymorphisms associated with pain sensitivity and temporomandibular joint disorders. *Methods in Molecular Biology*.

[9] Ahlers SJ, Elens LL, van Gulik L (2013). The Val158Met polymorphism of the COMT gene is associated with increased pain sensitivity in morphine treated-patients undergoing a painful procedure after cardiac surgery. *British Journal of Clinical Pharmacology*.

[10] Landau R, Liu SK, Blouin JL, Carvalho B (2013). The effect of OPRM1 and COMT genotypes on the analgesic response to intravenous fentanyl labor analgesia. *Anesthesia & Analgesia*.

[11] Zubieta J-K, Heitzeg MM, Smith YR (2003). COMT val158 genotype affects *μ*-opioid neurotransmitter responses to a pain stressor. *Science*.

[12] Berthele A, Platzer S, Jochim B (2005). *COMT* Val^108/158^Met genotype affects the mu-opioid receptor system in the human brain: evidence from ligand-binding, G-protein activation and preproenkephalin mRNA expression. *NeuroImage*.

[13] Diatchenko L, Slade GD, Nackley AG (2005). Genetic basis for individual variations in pain perception and the development of a chronic pain condition. *Human Molecular Genetics*.

[14] Nackley AG, Shabalina SA, Tchivileva IE (2006). Human catechol-*O*-methyltransferase haplotypes modulate protein expression by altering mRNA secondary structure. *Science*.

[15] Bialecka M, Kurzawski M, Klodowska-Duda G, Opala G, Tan E-K, Drozdzik M (2008). The association of functional catechol-*O*-methyltransferase haplotypes with risk of Parkinsons disease, levodopa treatment response, and complications. *Pharmacogenetics and Genomics*.

[16] Nackley AG, Shabalina SA, Lambert JE (2009). Low enzymatic activity haplotypes of the human catechol-*O*-methyltransferase gene: enrichment for marker SNPs. *PLoS ONE*.

[17] Novick D, Montgomery W, Kadziola Z (2013). Do concomitant pain symptoms in patients with major depression affect quality of life even when taking into account baseline depression severity?. *Patient Prefer Adherence*.

[18] Rana AQ, Depradine J (2011). Abdominal pain: a symptom of levodopa end of dose wearing off in Parkinson’s disease. *The West Indian Medical Journal*.

[19] Vela L, Lyons KE, Singer C, Lieberman AN (2007). Pain-pressure threshold in patients with Parkinson’s disease with and without dyskinesia. *Parkinsonism and Related Disorders*.

[20] Ford B (2010). Pain in Parkinson’s disease. *Movement Disorders*.

[21] Hatzimanolis A, Vitoratou S, Mandelli L (2013). Potential role of membrane-bound COMT gene polymorphisms in female depression vulnerability. *Journal of Affective Disorders*.

[22] Weintraub D, Comella CL, Horn S (2008). Parkinson’s disease—Part 1: pathophysiology, symptoms, burden, diagnosis, and assessment. *American Journal of Managed Care*.

[23] Alves G, Forsaa EB, Pedersen KF, Dreetz Gjerstad M, Larsen JP (2008). Epidemiology of Parkinson’s disease. *Journal of Neurology*.

[24] Lee MA, Walker RW, Hildreth TJ, Prentice WM (2006). A survey of pain in idiopathic Parkinson’s disease. *Journal of Pain and Symptom Management*.

[25] Białecka M, Kurzawski M, Roszmann A (2012). Association of COMT, MTHFR, and SLC19A1 (RFC-1) polymorphisms with homocysteine blood levels and cognitive impairment in Parkinson’s disease. *Pharmacogenet Genomics*.

[26] Yoshii F (2012). Pain and sensory disturbance in Parkinson disease. *Brain and Nerve*.

[27] Nègre-Pagès L, Regragui W, Bouhassira D, Grandjean H, Rascol O (2008). Chronic pain in Parkinson’s disease: the cross-sectional French DoPaMiP survey. *Movement Disorders*.

[28] Zambito Marsala S, Tinazzi M, Vitaliani R (2011). Spontaneous pain, pain threshold, and pain tolerance in Parkinson’s disease. *Journal of Neurology*.

[29] Letro GH, Quagliato EMAB, Viana MA (2009). Pain in Parkinson’s disease. *Arquivos de Neuro-Psiquiatria*.

[30] Hanagasi HA, Akat S, Gurvit H, Yazici J, Emre M (2011). Pain is common in Parkinson’s disease. *Clinical Neurology and Neurosurgery*.

[31] Chou K-L (2007). Reciprocal relationship between pain and depression in older adults: evidence from the English Longitudinal Study of Ageing. *Journal of Affective Disorders*.

[32] Graff-Guerrero A, Pellicer F, Mendoza-Espinosa Y, Martínez-Medina P, Romero-Romo J, de la Fuente-Sandoval C (2008). Cerebral blood flow changes associated with experimental pain stimulation in patients with major depression. *Journal of Affective Disorders*.

[33] Xia H, Wu N, Su Y (2012). Investigating the genetic basis of theory of mind (ToM): the role of catechol-*O*-methyltransferase (COMT) gene polymorphisms. *PLoS ONE*.

